# Association of lipoprotein(a) with ASCVD risk in women by menopausal status: the UK Biobank

**DOI:** 10.1016/j.ajpc.2026.101465

**Published:** 2026-02-06

**Authors:** Mikaila P. Reyes, Alexander C. Razavi, Harpreet S. Bhatia

**Affiliations:** a University of California San Diego School of Medicine, San Diego, CA, USA; b Division of Cardiology, Emory University School of Medicine, Atlanta, GA, USA; c Division of Cardiovascular Medicine, University of California San Diego, CA, USA

**Keywords:** Lipoprotein(a), Lipids, Prevention, Menopause, Risk factors

## Introduction

Atherosclerotic cardiovascular disease (ASCVD) is the leading cause of morbidity and mortality among women [1], with the onset of ASCVD often overlapping with the menopausal transition [2]. Altered lipoprotein metabolism during menopause is one contributing factor to increased ASCVD risk and is well-understood through increased LDL-cholesterol, increased triglycerides, and lower HDL-cholesterol [3]. However, very little is known regarding the role of lipoprotein(a) [Lp(a)] in menopause-associated ASCVD risk despite menopause being among few acquired conditions that may lead to an increase in Lp(a) [4]. Here, we sought to evaluate the association between Lp(a) and ASCVD events among women with and without menopause over an extended follow-up period.

## Methods

### Study design

The UK Biobank is a prospective, population-based cohort study of over 500,000 participants between the ages of 40 to 69 years. Participants were recruited between 2006 and 2010 across 22 assessment centers in the United Kingdom [5].

Serum Lp(a) was measured from blood samples taken during baseline assessment and analyzed using an immunoturbidimetric bioassay (Randox Biosciences, UK) with an analytical range of 3.8–189 nmol/L [6]. Menopause status was self-reported, based on if participants answered “Yes” to the question “Have you had your menopause (periods stopped)?”. Additional definitions of menopause used in sensitivity analyses are presented in Supplemental Table S1. The primary outcome was a composite of incident myocardial infarction (MI), incident hemorrhagic or ischemic stroke, or cardiovascular death.

### Statistical analysis

Only women were included in the present study (*n* = 273,297). Participants were excluded if they withdrew consent (*n* = 258), were missing Lp(a) measurement (*n* = 69,004), were missing co-variates of interest (*n* = 31,432), reported hormone replacement therapy (HRT) within 1 year (*n* = 15,654), or had a history of prior MI, stroke, or coronary artery bypass graft surgery (*n* = 3059). A total of 153,890 participants were included in the present analysis.

Baseline characteristics were stratified by menopause status and Lp(a) level (<125 nmol/L or ≥125 nmol/L and <75 nmol/L or ≥75 nmol/L). Continuous variables were compared using ANOVA or Kruskal-Wallis testing, as appropriate, while categorical variables were compared using Chi-Square testing.

**Fig. 1:**
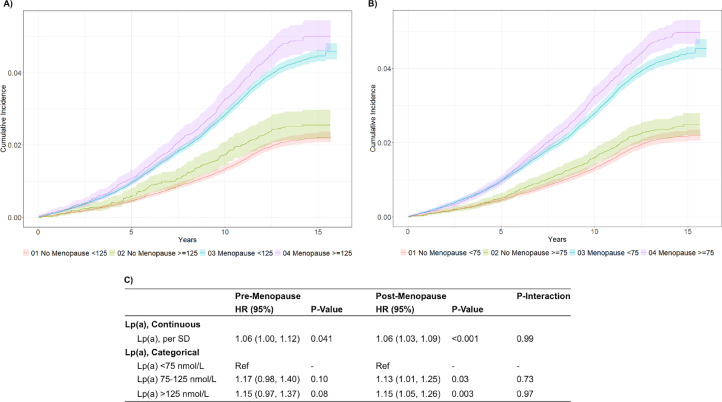
Lp(a) and ASCVD risk by menopausal status. (A) Unadjusted cumulative incidence of ASCD events by menopause status, Lp(a) ≥ or < 125 nmol/L. (B) Unadjusted cumulative incidence of ASCD events by menopause status, Lp(a) ≥ or < 75 nmol/L. (C) Multivariable association between Lp(a) and ASCVD according to menopausal status. Menopause Definition: Answered “Yes” to “Have you had your menopause (periods stopped)?” Models Adjusted For: age, race, smoking status, total cholesterol, HDL-C, cholesterol-lowering medication use, antihypertensive medication use, SBP, self-reported diabetes, glucose-lowering medication use, eGFR.

Cox proportional hazard models were used to evaluate the association between Lp(a) levels and incident ASCVD events, stratified by menopause status. Models were adjusted for traditional ASCVD risk factors, as noted in Fig. 1C. Lp(a) was evaluated continuously per standard deviation and as a categorical variable (low: <75 nmol/L, intermediate: 75–125 nmol/L, high: >125 nmol/L). For each model, the multiplicative interaction between Lp(a) and menopause status was evaluated. Sensitivity analyses were conducted in the same manner.

All statistical analyses were performed using R (version 4.4.1). Statistical significance was determined by a two-tailed p-value <0.05. The present study was deemed exempt from review by the University of California San Diego Human Research Protection Program (HRPP).

## Results

The average age of study participants was 56.1 years with 60.4 % of women reporting experiencing menopause. Median Lp(a) level was 22.5 [10.1, 62.1] nmol/L, with 21.4 % and 11.5 % of participants having Lp(a) ≥75 and ≥125 nmol/L, respectively. Median Lp(a) levels were higher among post-menopausal women (23.75; IQR [10.50, 61.60] nmol/L) than pre-menopausal women (20.79; IQR [9.47, 63.00] nmol/L) (*p* < 0.001). There were 5160 (3.4 %) incident ASCVD events over a median follow-up time of 13.7 years. Fig. 1A and 1B show the unadjusted cumulative incidence of ASCVD events stratified by menopause status and Lp(a) thresholds of 125 nmol/L and 75 nmol/L, respectively.

Each standard deviation increase in Lp(a) was associated with a 6 % higher risk of ASCVD among both pre- (HR 1.06, 95 % CI 1.00–1.12, *p* = 0.041) and post-menopausal (HR 1.06, 95 % CI 1.03–1.09, *p* < 0.001) women, with no significant interaction between Lp(a) level and menopausal status. Among both pre-menopausal and post-menopausal women, higher Lp(a) risk category was associated with increased ASCVD risk, but this only reached statistical significance among post-menopausal women. Post-menopausal women with Lp(a) levels of 75–125 nmol/L had a 13 % increase in risk of ASCVD events (HR 1.13, 95 % CI 1.01–1.25, *p* = 0.028) and those with Lp(a) levels greater than 125 nmol/L had a 15 % increase in risk of ASCVD events (HR 1.15, 95 % CI 1.05–1.26, *p* = 0.003), when compared to post-menopausal women with Lp(a) <75 nmol/L. Again, no significant interaction between Lp(a) category and menopausal status was noted (Fig. 1C).

In sensitivity analyses with different menopause definitions, similar associations were observed for Lp(a) level and ASCVD risk and no significant interactions were observed between menopause status and Lp(a) levels (Supplemental Table S2).

## Discussion

Among >150,000 women without a prior ASCVD event from the UK Biobank, higher Lp(a) levels were independently associated with increased ASCVD risk among both pre- and post-menopausal women. Consistent results were noted when Lp(a) was evaluated continuously or categorically, and no significant interaction between Lp(a) and menopausal status for ASCVD risk was noted. Despite higher median Lp(a) levels and incidence of ASCVD events among post-menopausal women, menopause status did not significantly modify the association between Lp(a) and ASCVD risk.

Our findings build on data from the Women’s Health Study by Ridker et al. which demonstrated that among 27,939 healthy women with a median age of 54.7 years at baseline and 30-year follow-up period, women with the highest quintile of Lp(a) levels (>44.1 mg/dL) had a significantly increased incidence of major adverse cardiovascular events when compared to the lowest quintile (<3.6 mg/dL) [7]. Similarly, Honigberg et al. demonstrated that in among 88,266 post-menopausal women in the UK Biobank study, women in the highest (61.50–189 nmol/L) vs lowest (3.80–10.31 nmol/L) Lp(a) quartiles had the highest risk of CHD [8]. The differences in the magnitude of the association between incident cardiovascular events in the highest vs lowest Lp(a) categories in Ridker et al. (HR 1.33), Honigberg et al. (HR 1.14), and our study (HR 1.15) are likely due to differences in Lp(a) categorization.

Although higher median Lp(a) levels were observed in post-menopausal women (23.75 nmol/L) than pre-menopausal women (20.79 nmol/L) in our study, definitive conclusions cannot be made regarding within-person changes in Lp(a) through the menopausal transition, as age alone has also been found to be associated with higher Lp(a) levels [9].

There were several strengths of the current study. First, our analysis adds to the current literature by assessing the interaction between menopause and Lp(a) on ASCVD risk in a large cohort. Secondly, the present findings re-emphasize the utility of Lp(a) screening in assessing ASCVD risk, with consideration of repeat Lp(a) testing among post-menopausal women who had intermediate Lp(a) levels prior to the menopausal transition[4].

The present findings should be interpreted in context of certain limitations. First, as an analysis of an observational cohort study, the data is subject to recall bias and missingness, which may result in misclassification of menopause status and outcome ascertainment. However, our findings were consistent across several different definitions of menopause. Due to the observational nature of the present study, there is also a potential for overadjustment for covariates which may mediate rather than confound the relationship between Lp(a) and ASCVD risk, attenuating differential associations in pre- and post-menopausal women. Second, the present study included women who had used, but discontinued HRT use >1 year prior to baseline assessment, which may have attenuated differences in Lp(a) levels even after discontinuation as post-menopausal women were more likely to have reported any HRT use. Third, UK Biobank participants tended to be healthier than the general population of the United Kingdom [10], increasing the possibility of healthy volunteer bias. Participants were also predominantly White, limiting generalizability to other racial/ethnic groups and suggesting the importance of validating the present findings in more diverse cohorts.

In conclusion, Lp(a) is an independent risk factor for incident ASCVD events among pre- and post-menopausal women, and menopausal status does not modify this association.

## Funding

Dr. Bhatia is supported by 10.13039/100000002National Institutes of Health, Grant 1K08HL166962 and UC San Diego BEACON. Dr. Razavi is supported by the 10.13039/100000050National Heart, Lung, and Blood Institute Grant L30HL175751.

## Disclosures

Dr. Bhatia - consultant / advisor for Kaneka, Novartis, NewAmsterdam, Arrowhead and Abbott.

## Ethical review statement

Participants provided informed consent upon recruitment for the UK Biobank study. The present study was deemed exempt from review by the University of California San Diego Human Research Protection Program (HRPP).

## Author agreement

We wish to draw the attention of the Editor to the following facts which may be considered as potential conflicts of interest and to significant financial contributions to this work.

We confirm that the manuscript has been read and approved by all named authors and that there are no other persons who satisfied the criteria for authorship but are not listed. We further confirm that the order of authors listed in the manuscript has been approved by all of us.

We confirm that we have given due consideration to the protection of intellectual property associated with this work and that there are no impediments to publication, including the timing of publication, with respect to intellectual property. In so doing we confirm that we have followed the regulations of our institutions concerning intellectual property.

We understand that the Corresponding Author is the sole contact for the Editorial process (including Editorial Manager and direct communications with the office). He/she is responsible for communicating with the other authors about progress, submissions of revisions and final approval of proofs. We confirm that we have provided a current, correct email address which is accessible by the Corresponding Author.

## CRediT authorship contribution statement

**Mikaila P. Reyes:** Writing – review & editing, Writing – original draft, Investigation, Formal analysis, Data curation. **Alexander C. Razavi:** Writing – review & editing, Writing – original draft, Methodology, Conceptualization. **Harpreet S. Bhatia:** Writing – review & editing, Supervision, Methodology, Conceptualization.

## Declaration of competing interest

The authors declare the following financial interests/personal relationships which may be considered as potential competing interests: Harpreet S. Bhatia reports financial support was provided by 10.13039/501100020300National Institutes of Health. Alexander C. Razavi reports financial support was provided by 10.13039/100000050National Heart Lung and Blood Institute. Harpreet S. Bhatia reports financial support was provided by 10.13039/100005522University of California San Diego. Harpreet S. Bhatia reports a relationship with Kaneka that includes: consulting or advisory. Harpreet S. Bhatia reports a relationship with Novartis that includes: consulting or advisory. Harpreet S. Bhatia reports a relationship with NewAmsterdam that includes: consulting or advisory. Harpreet S. Bhatia reports a relationship with Arrowhead that includes: consulting or advisory. Harpreet S. Bhatia reports a relationship with Abbott that includes: consulting or advisory. If there are other authors, they declare that they have no known competing financial interests or personal relationships that could have appeared to influence the work reported in this paper.

## Appendix Supplementary materials

Image, application 1
