# Supplementary material for: Association of lipoprotein(a) with ASCVD risk in women by menopausal status: the UK Biobank
Source: Am J Prev Cardiol. 2026 Feb 6;26:101465. doi: 10.1016/j.ajpc.2026.101465 (PMC13084097; doi:10.1016/j.ajpc.2026.101465)
Supplement: Supplementary file 1 [file mmc1.docx]

# SUPPLEMENTAL MATERIALS

| **Table S1: Menopause Definitions** | | |
| --- | --- | --- |
| **Analysis** | **Menopausal Defined As:** | **Pre-Menopausal Defined As:** |
| **01: Self-Reported Menopause (Primary Analysis)** | - Response to “Have you had your menopause (periods stopped)?” is “Yes” | - Response to “Have you had your menopause (periods stopped)?” is: - “No” - “Not Sure – Had a Hysterectomy” - “Not Sure – Other Reason” - “Prefer not to Answer” - Blank |
| **01A: Self-Reported Menopause, Excluding Women Unsure of Menopause Status** | - Response to “Have you had your menopause (periods stopped)?” is “Yes” | - Response to “Have you had your menopause (periods stopped)?” is “No” |
| **01B: Self-Reported Menopause, Including Women with Prior ASCVD Events** | - Response to “Have you had your menopause (periods stopped)?” is “Yes” | - Response to “Have you had your menopause (periods stopped)?” is: - “No” - “Not Sure – Had a Hysterectomy” - “Not Sure – Other Reason” - “Prefer not to Answer” - Blank |
| **02: Self-Reported Menopause or Bilateral Oophorectomy** | ANY of the following are true:   - Response to “Have you had your menopause (periods stopped)?” is “Yes” - Operation Code contains “hysterectomy with oophorectomy” - Operation Code contains “bilateral oophorectomy” - Response to "Have you had BOTH ovaries removed?" is “Yes” |  |
| **03: Self-Reported Menopause or Bilateral Oophorectomy without Hysterectomy** | ANY of the following are true:   - Response to “Have you had your menopause (periods stopped)?” is “Yes” - Operation Code contains “bilateral oophorectomy” - Response to "Have you had BOTH ovaries removed?" is “Yes”   AND *none* of the following are true:   - Operation Code contains “hysterectomy with cervical sparing” - Operation Code contains “hysterectomy with oophorectomy” - Response to “Have you had a hysterectomy (womb removed)?” is “Yes” - Response to “Have you had your menopause (periods stopped)?” is “Not sure – had a hysterectomy” |  |
| **04: Self-Reported Menopause, Bilateral Oophorectomy, or Hysterectomy** | ANY of the following are true:   - Response to “Have you had your menopause (periods stopped)?” is “Yes” - Response to "Have you had BOTH ovaries removed?" is “Yes” - Response to “Have you had your menopause (periods stopped)?” is “Not sure – had a hysterectomy” - Operation Code contains “bilateral oophorectomy” - Operation Code contains “hysterectomy with cervical sparing” - Operation Code contains “hysterectomy with oophorectomy” |  |

| **Supplemental Table S2:** Multivariable Association between Lp(a), per SD and ASCVD According to Menopausal Status, using Various Menopause Definitions | | | | | | |
| --- | --- | --- | --- | --- | --- | --- |
| **Menopause Definition** | | **Pre-Menopause** | | **Post-Menopause** | | **P-Interaction** |
|  | | **HR (95%)** | **P-Value** | **HR (95%)** | **P-Value** |  |
| **01A** | | 1.06 (0.96, 1.16) | 0.24 | 1.06 (1.03, 1.09) | <0.001 | 0.99 |
| **01B** | | 1.06 (1.01, 1.12) | 0.023 | 1.06 (1.03, 1.09) | <0.001 | 0.99 |
| **02** | | 1.06 (1.00, 1.13) | 0.046 | 1.05 (1.02, 1.08) | <0.001 | 0.69 |
| **03** | | 1.06 (1.02, 1.11) | 0.009 | 1.05 (1.02, 1.09) | 0.0014 | 0.84 |
| **04** | | 1.06 (0.97, 1.15) | 0.19 | 1.06 (1.03, 1.09) | <0.001 | 0.89 |
| **Models Adjusted For:** age, race, smoking status, total cholesterol, HDL-C, cholesterol-lowering medication use, antihypertensive medication use, SBP, self-reported diabetes, glucose-lowering medication use, eGFR  **Menopause Definitions:** 01A: Self-Reported Menopause, Excluding Women Unsure of Menopause Status; 01B: Self-Reported Menopause, Including Women with Prior ASCVD Events; 02: Self-Reported Menopause or Bilateral Oophorectomy; 03: Self-Reported Menopause or Bilateral Oophorectomy without Hysterectomy; 04: Self-Reported Menopause, Bilateral Oophorectomy, or Hysterectomy | | | | | | |
|  |  |  |  |  |  |  |
